# Mapping suicidal behaviour within transdiagnostic emotional systems: a network analysis of at-risk adolescents

**DOI:** 10.3389/fpsyg.2026.1918211

**Published:** 2026-09-30

**Authors:** Asier Pérez-Barrero, Alfonso Pérez-Esteban, Adriana Díez-Gómez, Alicia Pérez-Albéniz, Susana Al-Halabí, Beatriz Lucas-Molina, Felix Inchausti, Eduardo Fonseca-Pedrero

**Affiliations:** 1 Faculty of Health Sciences, Public University of Navarra, Pamplona, Spain; 2 Department of Educational Sciences, University of La Rioja, Logroño, Spain; 3 Department of Psychology, University of Oviedo, Oviedo, Spain; 4 Department of Developmental and Educational Psychology, University of Valencia, Valencia, Spain; 5 Department of Mental Health, Psychotherapy Research and Practice Unit, San Pedro University Hospital, Logroño, Spain

**Keywords:** adolescence, anxiety, depression, network analysis, suicidal behaviour, transdiagnostic dimensions

## Abstract

**Introduction:**

Suicidal behaviour is a major public health concern in adolescence. Traditional risk-factor models have shown limited predictive value, highlighting the need for approaches that better capture how emotional processes co-occur and relate to suicide risk. Transdiagnostic frameworks and network analysis may help clarify the role of core emotional dimensions in adolescent suicidality. This study examined the associations between suicide-related thoughts and behaviours and the transdiagnostic dimensions proposed by Brown and Barlow in adolescents with subthreshold anxiety and depressive symptoms.

**Method:**

The sample comprised 476 adolescents (*M* = 13.77 years; 73.9% female) identified through a multicenter school-based screening study in Spain (*n =* 8,746). To assess transdiagnostic dimensions of emotional disorders and suicidal behaviours, participants completed the Multidimensional Emotional Disorder Inventory and the SENTIA scale. A psychometric network analysis based on regularized partial correlations was estimated. Separate networks were estimated for adolescents with and without endorsement of suicide-related indicators. A Network Comparison Test (NCT) was conducted to examine differences in network structure, global strength, and individual edge weights between groups.

**Results:**

In the estimated psychological network, intrusive cognitions, and experiential avoidance emerged as the most central nodes, whereas suicide-related thoughts and behaviours occupied a relatively peripheral position. Descriptive network comparisons suggested different configurations across groups: among adolescents endorsing suicide-related indicators, neurotic temperament showed the highest centrality, whereas among those without such indicators, centrality remained concentrated on intrusive cognitions and experiential avoidance. However, the NCT did not identify statistically significant differences in global network structure or global strength between groups.

**Conclusion:**

Suicidal behaviour appears to be embedded in a broader system of transdiagnostic emotional processes. Intrusive cognitions, experiential avoidance, and generalized negative affect may be associated with clinically relevant targets for prevention and intervention. Findings support dimensional and transdiagnostic approaches to understanding suicidal behaviours in adolescents at risk for emotional disorders, while highlighting the need for further research in samples with greater clinical severity.

## Introduction

Suicidal behaviour is one of the major areas of public health and social concern, with a significant global impact (GBD 2019 Diseases and Injuries Collaborators, 2020). The World Health Organization (WHO) has estimated that around 700,000 people worldwide take their own lives each year (World Health Organization, 2025). In recent years suicide has become one of the leading causes of unnatural death among young people (Lovero et al., 2023; World Health Organization, 2021). Among adolescents, recent meta-analyses have found lifetime and annual prevalences of suicidal ideation of 20.5% (95% CI: 17.7–23.6%) and 15.1% (95% CI: 13.6–16.8%) respectively; lifetime and annual prevalences of suicide planning of 9.8% (95% CI: 7.4–12.9%) and 8.9% (95% CI: 7.1–11.2%), respectively; and lifetime and annual prevalences of suicide attempts of 7.1% (95% CI: 6.1–8.2%) and 5.2% (95% CI: 4.3–6.3%), respectively (Van Meter et al., 2023). Furthermore, it is associated with psychosocial morbidity and a need for healthcare resources, resulting in substantial healthcare and economic costs. Despite this alarming epidemiological and societal burden, there is broad international consensus regarding the existence of effective resources and interventions for both the prevention and clinical management of suicidal behaviour (Mann et al., 2021; Pirkis et al., 2023; Wasserman et al., 2021).

Traditional approaches have mainly examined isolated risk factors associated with suicidal behaviour. However, despite decades of research, their predictive capacity remains modest (Belsher et al., 2019; Franklin et al., 2017). This limitation has motivated the emergence of theoretical models that conceptualize suicidal behaviour as a complex and dynamic process resulting from the interaction of multiple psychological mechanisms rather than the additive effect of independent risk factors (de Beurs et al., 2021; Nguyen et al., 2025). Within this framework, ideation-to-action theories propose that the emergence of suicidal ideation and the progression from ideation to suicide attempts represent partially distinct processes with different determinants (Klonsky et al., 2016, 2018). Models such as the Interpersonal Theory of Suicide (Joiner, 2005), the Integrated Motivational-Volitional Model (O’Connor, 2011), and the Three-Step Theory (Klonsky and May, 2015) converge on the importance of interactions among multiple psychological processes rather than single causal factors. Nevertheless, a more precise understanding of how these mechanisms interact remains necessary to improve prevention and clinical intervention (de Beurs et al., 2021), as identifying core psychological processes may contribute to earlier detection and more effective preventive efforts.

One methodological framework that aligns closely with this perspective is network analysis. Since its introduction into psychopathology research (Borsboom and Cramer, 2013), the network approach has provided an alternative framework for conceptualizing mental disorders as systems of mutually interacting symptoms rather than manifestations of an underlying latent disorder (Borsboom, 2017; Fonseca-Pedrero, 2018; Fonseca-Pedrero and Muñiz, 2025). Network analysis enables the identification of direct relationships among variables (e.g., symptoms, traits) and the estimation of their relative importance within the system (Briganti et al., 2024). The network approach to psychopathology conceptualises symptoms and behaviours as nodes in a network, with causal connections linking these elements and giving rise to the emergence and maintenance of psychological disorders (Borsboom, 2017; Borsboom and Cramer, 2013; Fonseca-Pedrero, 2018). Consequently, it has become an increasingly valuable approach for studying and understanding suicidal behaviour, as it captures the dynamic interplay among multiple interacting factors consistent with contemporary theories of suicide and clinical staging models (Al-Halabí and Fonseca-Pedrero, 2024; Graziano et al., 2021; Iorfino et al., 2019; Scott et al., 2013; Shah et al., 2020).

Alongside these methodological developments, transdiagnostic approaches have gained increasing attention as a means of understanding psychopathology beyond traditional diagnostic taxonomies (Dalgleish et al., 2020; Fusar-Poli et al., 2019; Roefs et al., 2022). Rather than focusing on disorder-specific symptoms, transdiagnostic models examine psychological processes that are shared across multiple disorders. Among these, the dimensional model proposed by Brown and Barlow (2009) represents one of the most influential conceptualizations of emotional disorders. The model identifies ten transdiagnostic dimensions—including neurotic temperament, positive temperament, depressive mood, autonomic arousal, somatic anxiety, social anxiety, intrusive cognitions, traumatic re-experiencing, and avoidance—that characterize common mechanisms involved in the development, maintenance, and treatment response of anxiety and depressive disorders (Brown and Barlow, 2009; Osma et al., 2022).

Importantly, Brown and Barlow’s model may offer several advantages for the study of suicidal behaviour. Unlike broader dimensional frameworks, such as HiTOP (Kotov et al., 2017) or RdoC (Insel et al., 2010), it operationalizes emotional psychopathology into clinically meaningful dimensions that can be directly assessed and potentially targeted through intervention. Rather than representing diagnostic categories, these dimensions reflect core emotional processes—including emotional vulnerability, cognitive intrusions, avoidance, and positive affect—that have individually been associated with suicidal behaviour (Hughes et al., 2019; Melhem et al., 2019; Soto-Sanz et al., 2019). Studying these dimensions simultaneously therefore provides an opportunity to examine how shared emotional processes interact within a broader transdiagnostic system, potentially identifying mechanisms that transcend traditional diagnostic boundaries.

This perspective may be particularly relevant during adolescence. Emotional disorders commonly emerge during this developmental period, when emotional regulation capacities are still maturing and vulnerabilities begin to consolidate (Solmi et al., 2022). Difficulties in emotion regulation have consistently been associated with a broad range of psychopathological outcomes, including suicidal behaviours (Benton et al., 2021; Brenning et al., 2022; Cludius et al., 2020; de Berardis et al., 2020; Flores-Kanter et al., 2019; Hatkevich et al., 2019; Swee et al., 2020). Recent evidence indicates that impairments in emotional awareness, impulse control, emotional clarity, and access to adaptive regulation strategies contribute to the emergence and persistence of suicidal ideation (Turton et al., 2021). From a functional perspective, suicidal thoughts and behaviours may even operate as maladaptive emotion regulation strategies aimed at escaping intolerable emotional states, becoming negatively reinforced over time (Coppersmith et al., 2023; Law et al., 2015). From this perspective, suicidal behaviour cannot be explained solely by the presence of a specific disorder, but rather by a complex interaction between emotional vulnerability, affective intensity, difficulties in identifying and tolerating emotions, and impulsivity (Turton et al., 2021) Consequently, examining transdiagnostic emotional dimensions during adolescence may facilitate the identification of early mechanisms underlying suicidal behaviour before the onset of fully developed mental disorders.

To the best of our knowledge, previous network analyses have not examined the relationships between expressions of suicidal behaviour and transdiagnostic emotional dimensions in adolescents with subthreshold emotional symptoms. Within this framework, the present study used network analysis to explore the pattern of associations between suicidal behaviour and the transdiagnostic dimensions of emotional disorders proposed by Brown and Barlow (2009) in adolescents with subthreshold emotional symptoms. We hypothesized that suicidal behaviour would show positive associations with neurotic temperament, depressed mood, intrusive cognitions, traumatic re-experiencing, and experiential avoidance, and negative associations with positive temperament. Additionally, we expected the network structure to vary according to the presence of suicidal behaviour, with potential differences in network connectivity patterns, edge strengths, and node centrality. In particular, dimensions related to negative affectivity, especially neurotic temperament, were expected to occupy more central positions in the network of adolescents reporting suicidal behaviour.

## Method

### Participants

The sample was drawn from a multicentre project (Fonseca-Pedrero et al., 2023a,b) conducted in various provinces across Spain. The screening included 8,746 students from 85 schools (private, state and vocational) across 532 different classrooms. The exclusion criteria were: (a) having been diagnosed with a mental disorder or alcohol and/or substance dependence disorder, (b) the presence of a high risk of suicidal behaviour, (c) having a medical illness or condition that prevents the individual from participating in psychological treatment, (d) receiving other psychological treatment whilst the study is ongoing, (e) increases and/or changes in participants’ medication if they are receiving pharmacological treatment during the study, and (f) failing to complete or completing incorrectly (e.g., acquiescence, randomised responses) the baseline or follow-up assessments.

Accordingly, the present study focused specifically on adolescents with subthreshold emotional symptoms, representing an at-risk but non-clinical population. This design allowed the examination of transdiagnostic emotional dimensions during an early stage of emotional vulnerability, prior to the presence of severe psychopathology or high suicide risk.

Of the 8,746 adolescents assessed in the screening phase, 476 students were selected for presenting subclinical emotional symptoms. Inclusion criteria for the adolescents were the following: (a) 12–18 years of age (with a small number of younger students included because they belonged to the same school grades); (b) showing a moderate score in both the PHQ-9 (Kroenke et al., 2001) and GAD-7 (Spitzer et al., 2006) (between 10 and 15 points); and (c) Spanish proficiency. The exclusion criteria were (a) having been diagnosed with a mental disorder or alcohol and/or substance dependence disorder and/or high risk of suicidal behaviours; (b) having a medical disease or condition; (c) receiving on-going psychological treatment; or (d) no informed consent. The mean age was 13.77 years (*SD* = 1.43), with an age range of 10 to 18 years. The age distribution was as follows: 10 years, *n* = 1; 11 years, *n* = 2; 12 years, *n* = 99; 13 years, *n* = 124; 14 years, *n* = 115; 15 years, *n* = 66; 16 years, *n* = 50; 17 years, *n* = 18; and 18 years, *n* = 1.

### Instruments

*The Multidimensional Emotional Disorder Inventory* (MEDI) (Rosellini and Brown, 2019): a self-report questionnaire that assesses the transdiagnostic dimensions of emotional problems based on the model proposed by Brown and Barlow (2009). It consists of 49 Likert-type items on a 9-point scale (0 = not at all characteristic of me; 8 = completely characteristic of me). It includes nine scales: neurotic temperament, positive temperament, depressed mood, autonomic arousal, somatic anxiety, social anxiety, intrusive cognitions, traumatic re-experiencing, and avoidance. The final version of the instrument did not include the mania dimension, due to the low prevalence and severity of the symptoms it represents (Rosellini and Brown, 2019). The validated Spanish version for adults (Osma et al., 2022) was administered. The MEDI has demonstrated adequate psychometric properties when applied to adolescents with subclinical symptoms (Pérez-Esteban et al., 2024).

*The Brief Adolescent Suicidal Behaviour Assessment Scale* (SENTIA-Brief) (Díez-Gómez et al., 2020, 2021): a screening scale to detect the presence of suicidal behaviour in the adolescent population. It consists of 5 dichotomous (yes/no) items designed to assess the presence or absence of thoughts of death, suicidal ideation, suicide planning, suicidal communication and suicide attempts. The SENTIA-Brief has shown satisfactory reliability and validity evidence when administered in non-clinical adolescents (Díez-Gómez et al., 2020, 2021).

*Patient Health Questionnaire-9* (PHQ-9) (Kroenke et al., 2001). This 9-item self-report measure assesses depressive symptoms using a 4-point Likert scale ranging from 0 (not at all) to 3 (nearly every day). Higher scores reflect greater depressive symptomatology. The PHQ-9 has demonstrated adequate psychometric properties in Spanish samples (Fonseca-Pedrero et al., 2023a,b).

*Generalized Anxiety Disorder-7* (GAD-7) (Spitzer et al., 2006). This 7-item self-report measure assesses anxiety symptoms on a 4-point Likert scale ranging from 0 (not at all) to 3 (nearly every day). Higher scores reflect greater anxiety severity. The GAD-7 has demonstrated adequate psychometric performance in Spanish adolescent samples (Casares et al., 2024).

*The Oviedo Infrequency Scale–Revised (INF-OV-R)* (Fonseca-Pedrero et al., 2012) was used to detect random, pseudo-random, or dishonest responses. It is a 10-item dichotomous (Yes/No) self-report instrument. Participants with two or more incorrect responses were excluded from the final sample.

### Procedure

The participants agreed to take part in a clinical trial, namely the Evidence-based Psychology in Educational Contexts (PSICE) study, a randomised controlled trial registered (ClinicalTrials.gov, Identifier: NCT05322642), to examine the effectiveness of the Unified Protocol for Transdiagnostic Treatment of Adolescents (UP-A) (Ehrenreich-May, 2018) in educational settings. The PSICE study was approved by the ethics committees of the Government of La Rioja and the University of La Rioja (CEImLAR, PI552). Further details of the PSICE study have been published elsewhere (Fonseca-Pedrero et al., 2023a,b).

With the exception of the MEDI, all instruments were administered collectively during the screening phase using personal computers, in groups of 10–30 students, during regular school hours and in classrooms specifically prepared for this purpose. Participants were considered at risk when they obtained a moderate score (between 10 and 15 points) on both the PHQ-9 and the GAD-7 during the initial screening phase — the same criterion described in the Participants section — and were subsequently invited to complete the MEDI 1 week later. This assessment was conducted individually using electronic devices, in smaller groups of 10–15 students, outside of class time, and in designated classrooms.

Participation in the study was voluntary and no incentives were provided. For participants under 16 years of age, written informed consent was obtained from parents or legal guardians. All participants were assured of the confidentiality of their responses and their right to withdraw at any time.

### Data analysis

Firstly, a network analysis was conducted to explore the relational structure between transdiagnostic dimensions of emotional disorders and suicidal behaviour. A graphical Gaussian model (GGM) was estimated based on regularised partial correlations using the graphical LASSO (gLASSO) algorithm, with the regularisation parameter selected using the extended Bayesian information criterion (EBICglasso). The network included the nine MEDI dimensions and the total SENTIA-Brief score, calculated as the sum of its five dichotomous items (range = 0–5) and analysed as a continuous indicator of suicidal behaviour. All variables included in this network were standardised prior to analysis. The EBIC gamma hyperparameter was set at 0.5 (default value), balancing model parsimony and data fit.

Secondly, separate networks were estimated for adolescents with and without suicidal behaviour. Consistent with the use of the SENTIA-Brief as a screening instrument, suicidal behaviour was operationalised as the presence of any positive response to items assessing suicidal ideation, suicide planning, suicidal communication, or suicide attempt (items 2–5). This binary operationalisation was intended to identify adolescents presenting any clinically relevant manifestation of suicidal behaviour, regardless of its specific expression. However, this definition encompasses heterogeneous manifestations of suicidality, ranging from suicidal ideation to suicide attempts, and therefore does not distinguish between different stages of the suicidal process. To compare the symptomatic organisation between the groups, a differential analysis of centrality profiles was conducted. The centrality indices examined included strength, closeness, betweenness, and expected influence. In addition, a formal Network Comparison Test (NCT) (van Borkulo et al., 2023) was conducted to evaluate differences in global network structure, global strength, and individual edge weights between the two groups.

Thirdly, to verify the stability and accuracy of the network, the R package bootnet was used (Epskamp et al., 2018). Given that the combination of sample size and number of nodes results in a considerable computational load, a bootstrap analysis was performed. Network accuracy was evaluated using nonparametric bootstrap procedures with 1,000 resamples. The stability of centrality indices was assessed using case-dropping bootstrap procedures and quantified using the correlation stability (CS) coefficients. Following Epskamp et al. (2018), CS > 0.25 are considered acceptable, with values above 0.50 indicating preferable stability. However, these thresholds should be interpreted with caution, as they are based on simulation results. The JASP programme (JASP Team, 2025) and the R environment (R Core Team, 2025) were used for the analyses.

## Results

### Network analysis of the associations between suicidal behaviour and transdiagnostic dimensions of emotional problems

The estimated psychometric network illustrating the relationships between suicidal behaviour and the transdiagnostic dimensions of emotional problems is shown in Figure 1. The estimated network revealed a complex pattern of positive and negative associations among the included variables, reflecting the interconnected nature of transdiagnostic emotional dimensions and suicidal behaviour.

**Figure 1:**
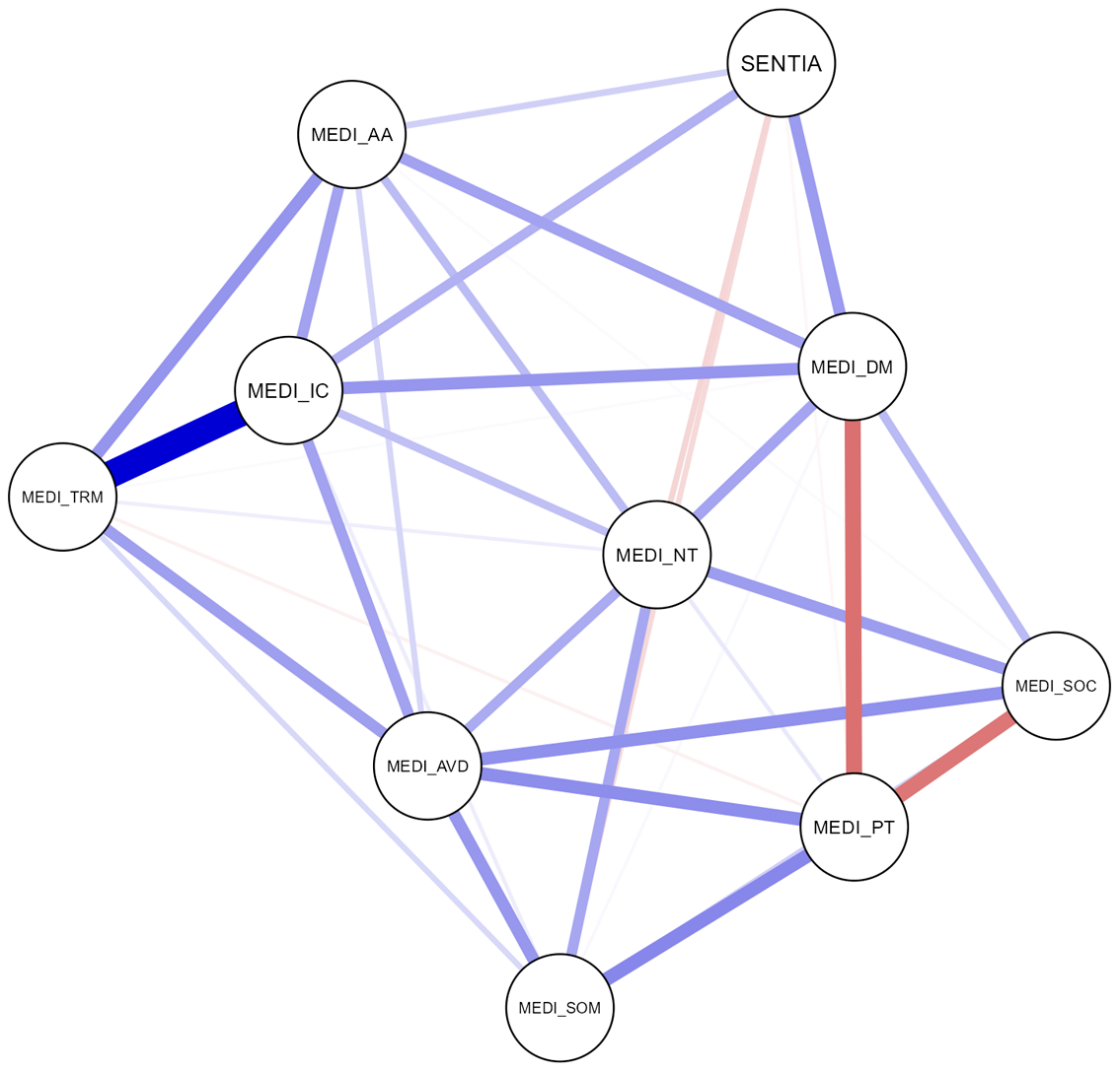
A network analysis of suicidal behaviour and transdiagnostic dimensions of emotional problems in a sample of adolescents with anxiety-depressive symptoms. MEDI_NT, neurotic temperament; MEDI_PT, positive temperament; MEDI_DM, depressed mood; MEDI_AA, autonomic arousal; MEDI_SOM, somatic anxiety; MEDI_SOC, social anxiety; MEDI_IC, intrusive cognitions; MEDI_TRE, traumatic re-experiencing; MEDI_AVD, avoidance. SENTIA, suicidal behaviour. The thicker the line, the stronger the relationship between nodes. A blue line indicates a positive relationship, whilst a red line indicates a negative relationship.

The strongest connections were observed between intrusive cognitions (IC) and post-traumatic re-experiencing (TRM), as well as between positive temperament (PT) and somatic anxiety (SOM), and between emotional/experiential avoidance (AVD) and social anxiety (SOC) (Table 1). Negative associations were also identified between PT and other dimensions, including depressive mood (DM) and social anxiety (SOC). IC and AVD presented the highest values for strength and expected influence, indicating that these dimensions were highly connected within the estimated network. In contrast, suicidal behaviour, PT, and SOC were among the nodes with the lowest centrality, suggesting a more peripheral role in the system of relationships.

**Table 1:** Network strength centrality indices using MEDI dimensions and the total SENTIA score.

Dimension	Betweenness	Closeness	Strength	Expected influence
AA	−0.802	−0.998	−0.669	0.262
AVD	0.630	0.786	1.084	1.196
DM	1.777	1.661	0.811	−0.158
IC	0.344	0.746	1.455	1.380
NT	−0.802	−0.037	0.293	0.450
PT	1.490	0.779	0.231	−1.845
SOC	−0.516	−0.582	−0.607	−0.797
SOM	−0.802	−0.864	−0.640	0.022
TRM	−0.516	0.050	−0.041	0.520
SENTIA	−0.802	−1.541	−1.919	−1.030

NT, Neurotic temperament; PT, Positive temperament; DM, Depressed mood; AA, Autonomic arousal; SOM, Somatic anxiety; SOC, Social anxiety; IC, Intrusive cognitions; TRM, Traumatic re-experiencing; AVD, Avoidance. SENTIA, Suicidal behaviour. The thicker the line, the stronger the relationship between nodes. A blue line indicates a positive relationship, whilst a red line indicates a negative relationship.

### Network analysis of transdiagnostic dimensions of emotional problems in adolescents with and without suicidal behaviour

Separate networks were estimated for participants with and without current suicidal behaviour, defined by affirmative responses to items 2–5 of the SENTIA-Brief scale (see Figures 2, 3). Both networks showed connected structures, with differences in the distribution of centrality indices (Figure 4). In the group without suicidal behaviour (*n* = 324), the most central dimensions according to the strength and expected influence indices were AVD and IC. A relatively high weight was also observed for PT in some indices such as strength and betweenness. In line with this, in terms of betweenness, AVD occupied a prominent position, indicating its potential role as a link between different symptom domains. The estimated network suggested a pattern of relatively stronger associations among AVD, IC, and SOM, with comparatively weaker connections involving physiological arousal (AA) and SOC.

**Figure 2:**
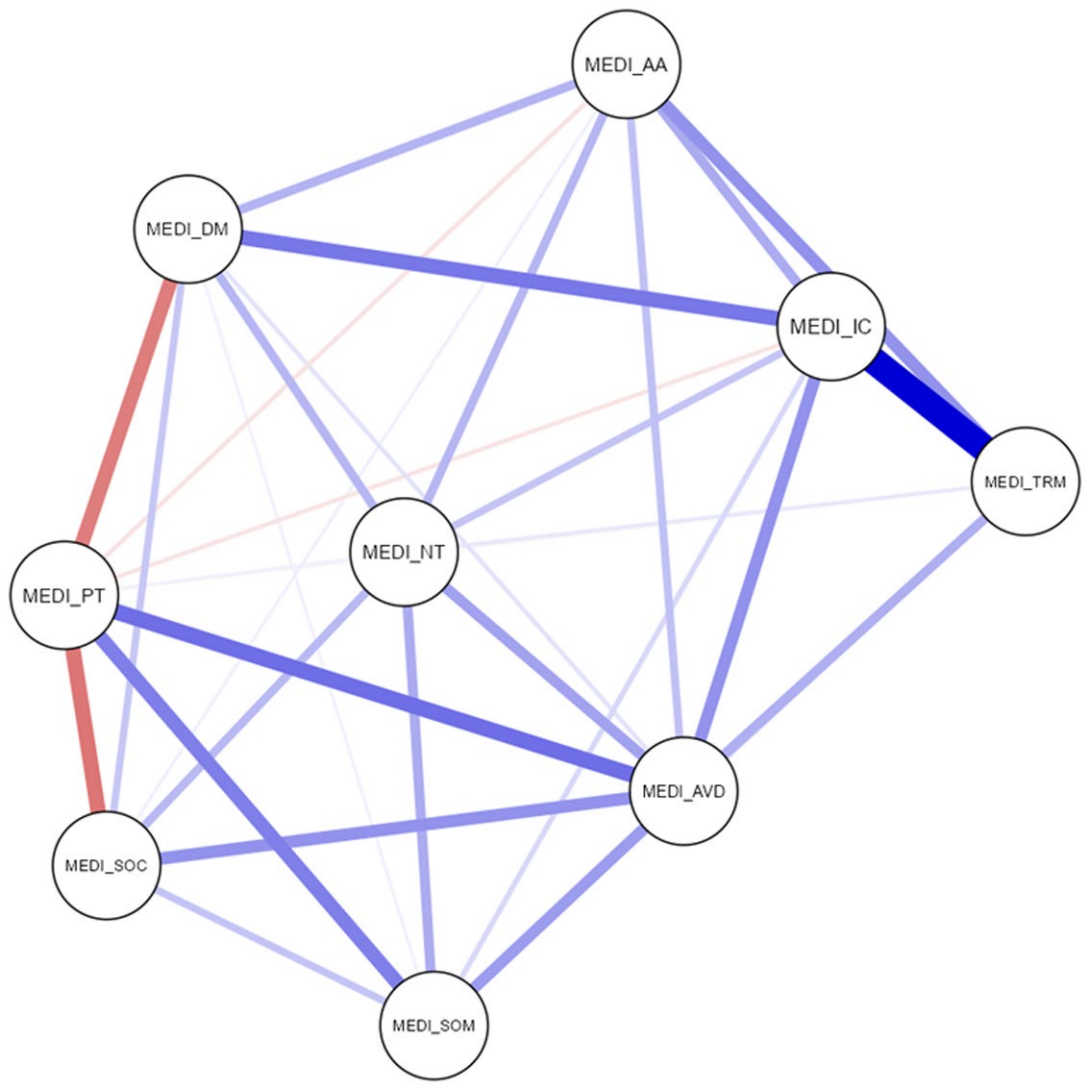
Estimated network of suicidal behaviour and transdiagnostic dimensions of emotional problems in the subsample of adolescents with anxiety-depressive symptoms and no suicidal ideation or behaviour. MEDI_NT, neurotic temperament; MEDI_PT, positive temperament; MEDI_DM, depressed mood; MEDI_AA, autonomic arousal; MEDI_SOM, somatic anxiety; MEDI_SOC, social anxiety; MEDI_IC, intrusive cognitions; MEDI_TRM, traumatic re-experiencing; MEDI_AVD, avoidance. SENTIA, suicidal behaviour. The thicker the line, the stronger the relationship between nodes. A blue line indicates a positive relationship, whilst a red line indicates a negative relationship.

**Figure 3:**
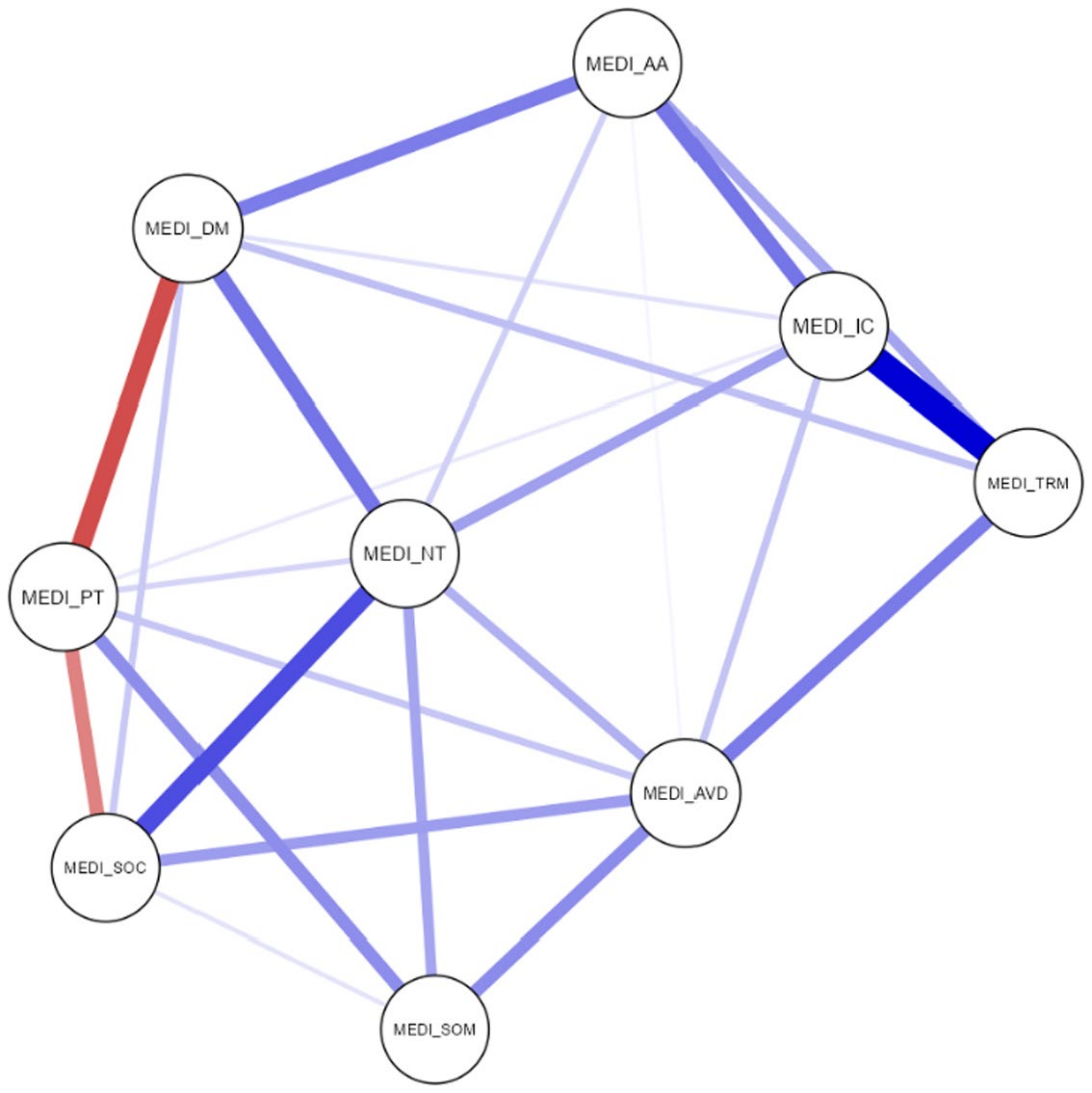
Estimated network of suicidal behaviour and transdiagnostic dimensions of emotional problems in the subsample of adolescents with suicidal ideation/behaviour and anxiety-depressive symptoms. MEDI_NT, neurotic temperament; MEDI_PT, positive temperament; MEDI_DM, depressed mood; MEDI_AA, autonomic arousal; MEDI_SOM, somatic anxiety; MEDI_SOC, social anxiety; MEDI_IC, intrusive cognitions; MEDI_TRM, traumatic re-experiencing; MEDI_AVD, avoidance. SENTIA, suicidal behaviour. The thicker the line, the stronger the relationship between nodes. A blue line indicates a positive relationship, whilst a red line indicates a negative relationship.

**Figure 4:**
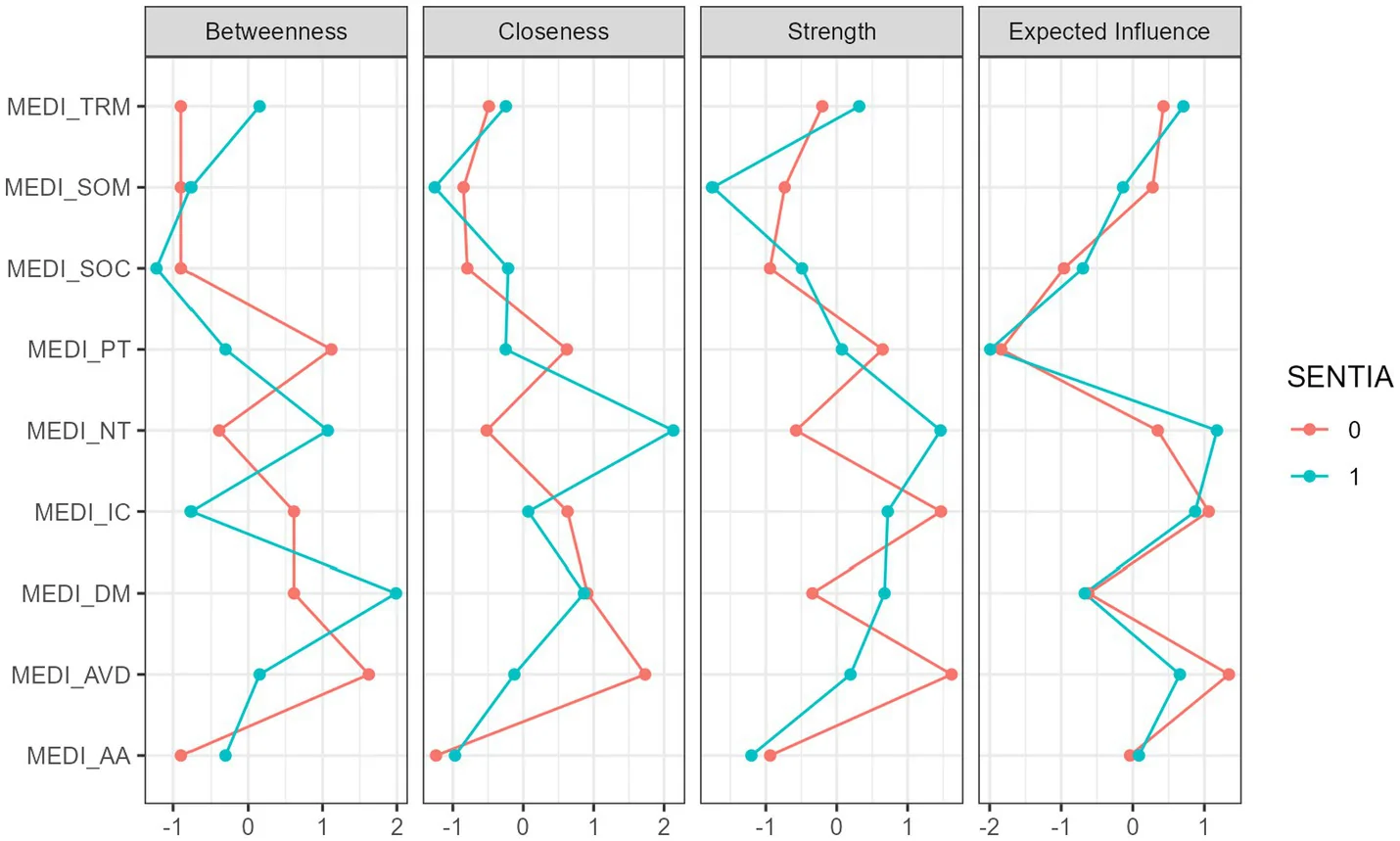
Strength centrality indices in samples with and without suicidal ideation/behaviour. MEDI_NT, neurotic temperament; MEDI_PT, positive temperament; MEDI_DM, depressed mood; MEDI_AA, autonomic arousal; MEDI_SOM, somatic anxiety; MEDI_SOC, social anxiety; MEDI_IC, intrusive cognitions; MEDI_TRM, traumatic re-experiencing; MEDI_AVD, avoidance. SENTIA, suicidal behaviour. The thicker the line, the stronger the relationship between nodes. A blue line indicates a positive relationship, whilst a red line indicates a negative relationship. Group 0 = no suicidal behaviour. Group 1: Suicidal behaviour present.

In the group with present suicidal ideation or behaviour (*n* = 152), neurotic temperament (NT) emerged as the most central node in almost all indices, followed by DM and IC. In the estimated network, AVD showed a considerable reduction in centrality relative to group without current suicidal ideation or behaviour. Furthermore, several dimensions—such as SOC, AA and SOM—showed negative or reduced values in the centrality indices, indicating lower integration within the psychological network.

### Network comparison test

The test revealed no statistically significant differences in overall network structure between adolescents with and without suicidal behaviour (*M* = 0.20, *p* = 0.406). Likewise, no statistically significant differences was found in global network strength (*S* = 0.05, *p* = 0.890), indicating comparable overall connectivity across groups.

Although several individual edges showed nominally significant differences (e.g., neurotic temperament–social anxiety, positive temperament–intrusive cognitions, depressed mood–intrusive cognitions, and depressed mood–traumatic re-experiencing), these findings should be interpreted cautiously given the large number of edge comparisons performed.

### Stability and accuracy of the estimated networks

The results of the bootstrap analysis indicated that the network was estimated with adequate accuracy. Edge weights showed narrow confidence intervals for the strongest connections and wider intervals for weaker ones, indicating good overall accuracy (Supplementary Figure S1). Correlation stability (CS) coefficients were 0.75 for strength, 0.59 for closeness, and 0.36 for betweenness, all exceeding the recommended minimum threshold of 0.25 (Epskamp et al., 2018), and reaching the preferred threshold of 0.50 for strength and closeness (Supplementary Figure S2). These results indicate that the centrality indices, particularly strength, can be interpreted with confidence.

## Discussion

To the best of our knowledge, this study constitutes the first network analysis to comprehensively examine the relationship between suicidal behaviour and the transdiagnostic dimensions of emotional problems in a sample of adolescents with subclinical anxiety-depressive symptoms. This work adds to the body of literature from recent years that has highlighted the explanatory and research potential of network theory in adolescent suicidal behaviour (Brown et al., 2021; Delgadillo et al., 2023; Fonseca-Pedrero et al., 2024; Gijzen et al., 2021; Graziano et al., 2021; Holman and Williams, 2022; Karnick et al., 2022). Furthermore, it forms part of efforts to move towards complex, dimensional and staging models that allow us to overcome the limitations of categorical, unidimensional and linear approaches to so-called suicide ‘risk’ (Hawton et al., 2022; Knipe et al., 2022). Taken together, the findings point to the value of conceptualising suicidal behaviour as a complex phenomenon resulting from the interaction between different biographical variables and transdiagnostic processes, and not merely as a passive consequence of a supposed latent common cause termed a ‘disorder’ (Borsboom, 2017; de Beurs, 2017).

Firstly, the dimensions of intrusive cognitions and emotional/experiential avoidance consistently emerged as the most central nodes, both in terms of strength and expected influence. The centrality of intrusive cognitions and emotional/experiential avoidance suggests that these processes may occupy relatively prominent positions within the estimated network (Gerdan and Salcioglu, 2025; Shaw et al., 2021; Zuzama et al., 2020). From a functional perspective, both processes are closely linked to maladaptive emotional regulation strategies (Coppersmith et al., 2023). Accumulating evidence suggests that rumination and cognitive intrusion contribute to the maintenance of negative affect, reduce cognitive flexibility, and hinder active problem-solving in both the general population and adolescents (Blanke et al., 2022; Watkins and Roberts, 2020; Zuzama et al., 2020). Complementarily, experiential avoidance (Hayes et al., 1996)—understood as the persistent attempt to escape, suppress or control aversive internal states—has been associated with a paradoxical increase in emotional distress and with more persistent psychopathological trajectories (Chiles et al., 2018; Fernández-Rodríguez et al., 2018; Gerdan and Salcioglu, 2025; Mellick et al., 2019; Wenze et al., 2018). The central position of these nodes in the estimated network suggests that intrusive cognitions and experiential avoidance may represent particularly relevant transdiagnostic processes in the organisation of emotional difficulties in this population. This interpretation is consistent with functional models of suicide that conceptualise suicidal ideation as a form of cognitive or emotional escape from affective states perceived as unbearable (Baumeister, 1990; Blacutt et al., 2025; Landrault et al., 2020).

In this study, suicidal behaviour occupied a peripheral position within the estimated global network. This finding may indicate that suicidal behaviour is less centrally connected within the estimated network and appears to be situated within a broader pattern of transdiagnostic emotional processes, at least in adolescents at risk of emotional disorders. This configuration is consistent with contemporary models that understand suicidal behaviour as an emergent result of the interaction between multiple psychological processes, rather than as a central phenomenon that reorganises the psycho(patho)logical system on its own (de Beurs et al., 2019, 2021; Fonseca-Pedrero et al., 2024).

Positive temperament (i.e., tendency to experience positive affect, social engagement, and goal-directed behaviour) occupied a clearly peripheral position in the global network, with negative associations with dimensions such as depressive mood and social anxiety, and low centrality indices. From a transdiagnostic framework, positive temperament is conceptualised as a relatively independent temperamental dimension, more closely related to processes of approach, motivation and reward than to threat and avoidance circuits (Barnes-Horowitz et al., 2024; Naragon-Gainey, 2019; Zhu et al., 2022). Its low integration into the network could be interpreted as a protective factor rather than a structural element of the symptomatic system. In this sense, positive affect does not appear to organise emotional distress, but it could modulate its impact, dampening the intensity of depressive mood, anxiety symptoms, stress levels (Barnes-Horowitz et al., 2024; Schürmann-Vengels et al., 2025; Taylor et al., 2017) or suicidal behaviour itself (Bennardi et al., 2019; Teismann et al., 2019). This finding may have clinical implications, as strengthening positive affect could contribute to improved emotional functioning; however, its influence within networks characterized by strong interconnections among avoidance and intrusion processes remains unclear.

Secondly, descriptive comparisons of the transdiagnostic networks suggested differences in the relative centrality of several dimensions according to the presence or absence of suicidal behaviour. However, the Network Comparison Test (NCT) revealed no statistically significant differences in overall network structure or global strength. Therefore, these findings should be interpreted as descriptive differences in the relative prominence of specific emotional dimensions rather than evidence of distinct underlying network architectures. Nonetheless, variations in node centrality may offer useful insights into how transdiagnostic emotional processes are organized across different subgroups of adolescents. In the group without suicidal behaviour, the estimated network is organised primarily around intrusive cognitions and emotional/experiential avoidance, maintaining a structure similar to that observed in the overall network. In this context, avoidance appears to play a coordinating role, linking different emotional dimensions and facilitating the persistence of psychological distress. Conversely, in the group with suicidal behaviour, Neurotic temperament showed the highest centrality values in the estimated network, both in terms of strength and expected influence. Although this difference in centrality should be interpreted cautiously in light of the non-significant difference in overall network structure, this finding may suggest that adolescents with suicidal behaviour exhibit a pattern of covariation among emotional variables in which a generalised negative affect predominates, characterised by high emotional reactivity, sensitivity to threat, and a persistent tendency to experience negative emotions (Naragon-Gainey, 2019; Zhu et al., 2022). This centrality of neuroticism is consistent with the hierarchical architecture of Brown and Barlow’s (2009) model underpinning the MEDI, where neuroticism constitutes a temperamental dimension—a stable variable related to the risk of developing emotional disorders— whilst the remaining dimensions of the MEDI are phenotypic and represent the contextual expression of this vulnerability (Rosellini and Brown, 2019). The predominance of neuroticism as a central node in the group with suicidal behaviour may indicate that the risk of emotional psychopathology is higher in this group, with neurotic temperament occupying a more central position within the symptomatic network (Barlow et al., 2014; Lawson et al., 2022; da Mota et al., 2024). As noted above, these centrality differences should be understood as descriptive rather than confirmatory.

Alongside neurotic temperament, cognitive intrusions played a central role in the estimated network of the group with suicidal behaviour, showing higher centrality indices than depressive mood. This pattern suggests that suicidal ideation may show stronger associations with cognitive intrusion processes (Cha et al., 2019; da Silva et al., 2018; Rutter et al., 2020) and general emotional distress than with depressive symptoms within the present network. Depressive mood, although connected to other relevant nodes, did not emerge as the sole dominant node in the network structure, suggesting that suicidal behaviour in adolescents cannot be explained exclusively by depressive symptoms (Melhem et al., 2019). A particularly relevant finding is the loss of centrality of experiential avoidance in the group with suicidal ideation. Although this difference should be interpreted as a difference in the relative centrality profile rather than as evidence of a statistically significant network reorganisation, one possible interpretation is that, in more advanced stages of the ideation–action continuum, avoidance strategies cease to be functional or sufficient for regulating distress, while diffuse negative affect and persistent cognitive intrusion become relatively more prominent. This reorganisation is consistent with ideation–action models (Klonsky et al., 2018), which propose differential mechanisms involved in the transition from suicidal ideation to the act of suicide itself (Kirshenbaum et al., 2024; Marie et al., 2020). Furthermore, the reduced integration of dimensions such as social anxiety, autonomic arousal or somatic anxiety within the network of the group exhibiting suicidal behaviour may reflect a less differentiated emotional configuration, in which affective experiences become increasingly dominated by diffuse and persistent negative affect (Naragon-Gainey, 2019; Zhu et al., 2022).

Beyond global network structure, the NCT identified nominally significant differences in four specific edges: neurotic temperament–social anxiety, positive temperament–intrusive cognitions, depressed mood–intrusive cognitions, and depressed mood–traumatic re-experiencing. Although these results should be interpreted with caution given the number of edge-level comparisons performed, they may suggest that adolescents with suicidal behaviour present a somewhat tighter coupling between generalized negative affect (neuroticism, depressed mood) (Naragon-Gainey, 2019; Zhu et al., 2022) and cognitive intrusion/re-experiencing processes (Blanke et al., 2022; Kralj et al., 2024), alongside a weaker buffering role of positive temperament (Barnes-Horowitz et al., 2024). This pattern is broadly consistent with the centrality shift towards neurotic temperament and intrusive cognitions described above, and warrants replication in larger, independent samples.

The findings of this study are consistent with the growing call for complex dynamic systems models, staging approaches, and transdiagnostic frameworks in the understanding and prevention of suicidal behaviour (Hayes and Andrews, 2020; Nelson et al., 2017; Rief et al., 2023). From this perspective, suicidal behaviour is not adequately explained by lists of isolated risk factors, but rather as the result of dynamic interactions between core processes (e.g., emotional dysregulation) depending on individuals’ life histories and contexts. In clinical terms, the findings highlight the potential relevance of targeting core processes such as experiential avoidance, intrusive cognitions and neurotic temperament, beyond specific symptom reduction, while recognising that centrality does not by itself establish causal importance or direct intervention targets. Transdiagnostic interventions (Barlow et al., 2011; Ehrenreich-May, 2018), as well as approaches based on acceptance of internal experience, emotional regulation and emotional exposure, may prove particularly relevant in adolescent populations at subclinical risk (Ehrenreich-May et al., 2017; Muhammad et al., 2026). Emerging evidence from more integrative and process-oriented interventions in adolescents also supports the clinical relevance of targeting these core mechanisms within a broader focus on personality functioning and interpersonal meaning-making. For instance, recent findings from Metacognitive Interpersonal Group Therapy for adolescents with personality disorders suggest that interventions addressing metacognition, interpersonal schemas, and emotional regulation can lead to meaningful improvements in psychological functioning in youths with more complex and severe clinical presentations (Inchausti et al., 2026). However, it must not be forgotten that suicidal behaviour is a complex and multifactorial phenomenon whose definition, assessment, prevention, intervention and postvention require a comprehensive approach focused on the individual’s sense of life and suffering within their biographical, social and cultural context avoiding overly reductionist interpretations (Al-Halabí and Fonseca-Pedrero, 2024; Besch et al., 2024).

This study has several limitations that must be considered. Firstly, the systematic exclusion from the PSICE study of adolescents at acute risk of suicide—implemented for ethical and safety reasons—creates a selection bias that limits the generalization of the findings to the population with greater clinical severity. It is possible that the configuration of the relationships between emotional variables in adolescents experiencing an acute suicidal crisis differs qualitatively from the patterns observed here. The results should therefore be interpreted as applicable to adolescents with subclinical anxiety-depressive symptoms and low- to moderate-intensity suicidal behaviour. Furthermore, the sample comes from a screening-based trial with specific inclusion criteria (subthreshold symptoms), which limits generalization to unselected clinical or community populations. However, this characteristic of the sample is consistent with early detection and clinical staging models, which emphasize the importance of identifying patterns of vulnerability in the early stages of the psychopathological continuum, before more severe and less reversible configurations become established (Cross et al., 2014; Fusar-Poli et al., 2021). Secondly, the cross-sectional design prevents the establishment of causal inferences regarding the relationships observed in the network. Moreover, although highly central nodes may help identify relevant transdiagnostic processes, centrality should not be interpreted as evidence of causal importance or direct intervention targets. Thirdly, the sample size of the subgroup with indicators of suicidal behaviour may be insufficient to ensure the stability of the network estimates, necessitating a cautious interpretation of the differences in centrality profiles between the two groups. Similarly, the relatively small subgroup may have limited the statistical power of the NCT to detect subtle differences in network structure or individual edge weights. Therefore, the absence of statistically significant differences in overall network structure and global strength should not be interpreted as definitive evidence of complete equivalence between groups. Fourthly, the use of self-reported measures may introduce response bias. Fifthly, the sample was predominantly female (73.9%), and sex/gender differences in network structure were not specifically examined. Therefore, the generalizability of the findings to adolescent boys and to more gender-balanced populations is limited. Finally, suicidal behaviour was assessed using a dichotomous total score and a self-administered scale at a single point in time, which limits both the differentiated analysis of the various components of the ideation-action continuum and the understanding of its dynamic and fluctuating nature. Furthermore, the operationalization of suicidal behaviour as the presence of any suicide-related indicator may have obscured potentially meaningful differences across stages of the ideation-to-action continuum.

The results of the present study contribute to understanding adolescent suicidal behaviour within a framework of interconnected transdiagnostic emotional processes. Although the subgroup analyses revealed differences in the relative centrality of several transdiagnostic dimensions, the NCT did not identify statistically significant differences in overall network structure or global strength between adolescents at risk for emotional disorders with and without suicidal behaviour. Thus, the findings suggest that suicidal behaviour may be associated with changes in the relative prominence of particular emotional processes within a broadly stable transdiagnostic architecture, rather than with a fundamentally different network organisation. These findings point towards a conceptualisation of suicidal behaviour that moves away from categorical and linear models towards dimensional and dynamic approaches, capable of capturing the complexity of human experience (Fusar-Poli et al., 2026). Within the framework of selective and indicated prevention, integrating the network perspective and the transdiagnostic approach into research and clinical practice may open up new opportunities for earlier, more integrated and personalised interventions, aimed at improving early detection and informing prevention efforts in adolescent populations.

## Data Availability

The raw data supporting the conclusions of this article will be made available by the authors, without undue reservation.
