# Supplementary material for: Mapping suicidal behaviour within transdiagnostic emotional systems: a network analysis of at-risk adolescents
Source: Front Psychol. 2026 Sep 30;17:1918211. doi: 10.3389/fpsyg.2026.1918211 (PMC13670509; doi:10.3389/fpsyg.2026.1918211)
Supplement: Supplementary file 1 [file Data_Sheet_1.PDF]

**Supplementary Material**

**Figure S1.** *Edge Weight Accuracy (Nonparametric Bootstrap)*

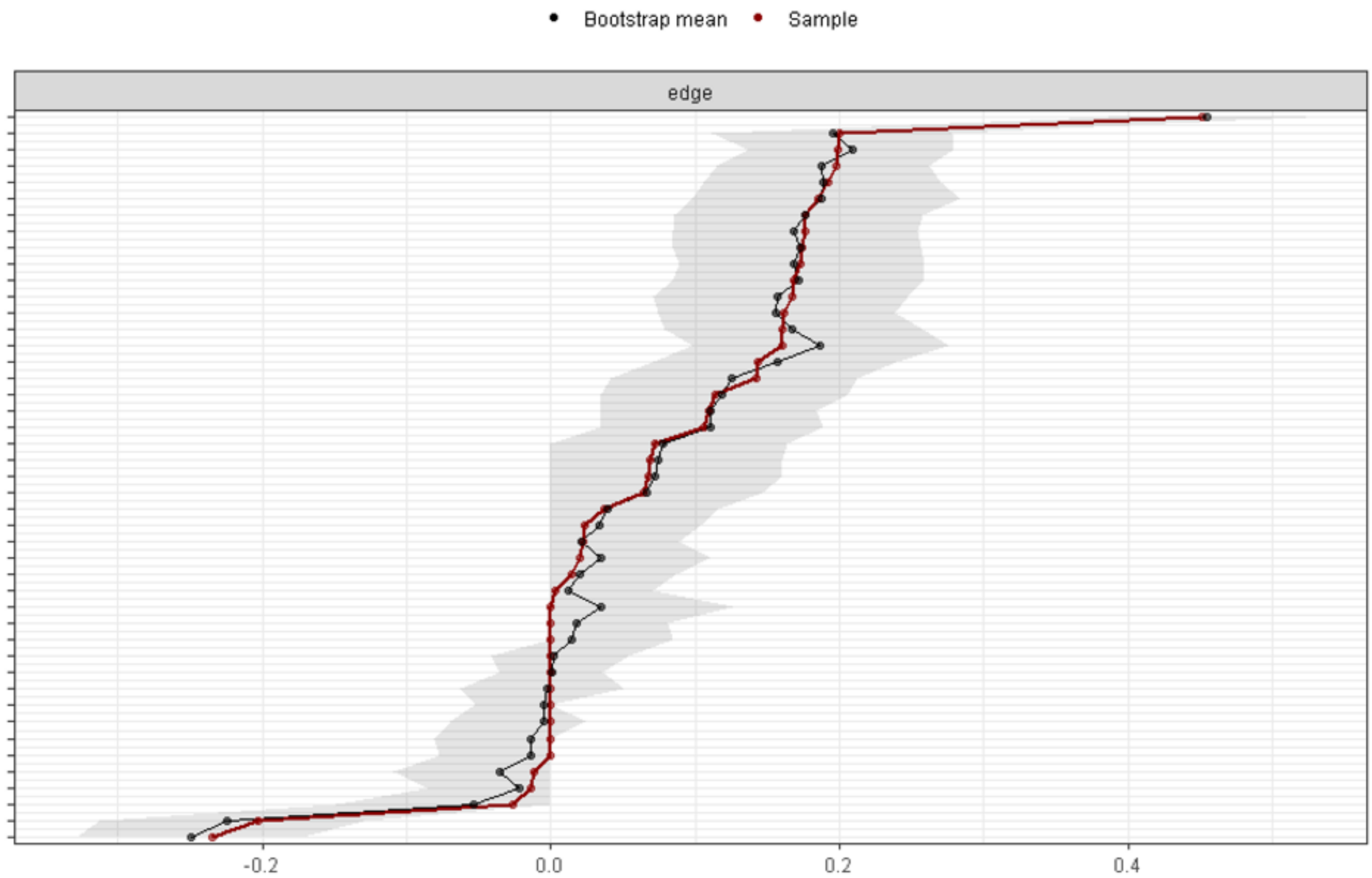


*Note:* Bootstrapped 95% confidence intervals for the estimated edge weights (nBoots = 1,000). Red dots indicate the edge weight in the original sample; black dots indicate the bootstrap mean; grey bands represent the 95% confidence interval. Narrower bands indicate more precisely estimated edges, while wider bands reflect greater uncertainty, typically observed for weaker connections.

**Figure S2.** *Stability of Centrality Indices (Case-Dropping Bootstrap)*

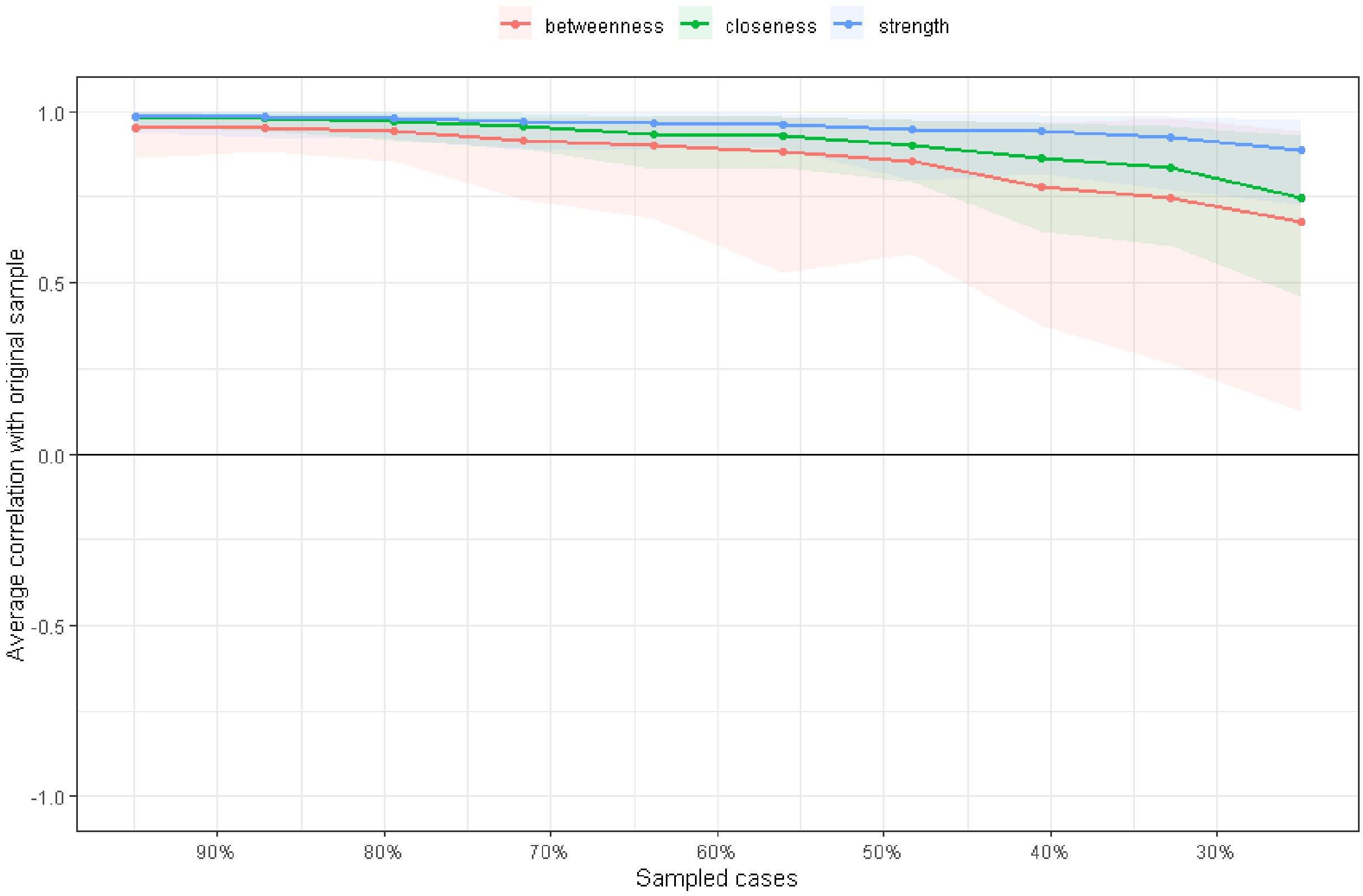


*Note:*Average correlation between centrality indices (strength, closeness, betweenness) computed on subsamples of increasingly reduced size and the indices from the full sample (nBoots = 1,000). Correlation stability (CS) coefficients were .75 for strength, .595 for closeness, and .361 for betweenness, all above the recommended minimum threshold of .25 (Epskamp et al., 2018).
